# Assessing beliefs about emotions: Development and validation of the Emotion Beliefs Questionnaire

**DOI:** 10.1371/journal.pone.0231395

**Published:** 2020-04-14

**Authors:** Rodrigo Becerra, David A. Preece, James J. Gross

**Affiliations:** 1 School of Psychological Science, The University of Western Australia, Perth, Western Australia, Australia; 2 School of Psychology, Curtin University, Perth, Western Australia, Australia; 3 Department of Psychology, Stanford University, Stanford, California, United States of America; University of Lleida, SPAIN

## Abstract

People’s beliefs about emotions may be grouped into two main categories: beliefs about the *controllability* of emotions and beliefs about the *usefulness* of emotions. These beliefs influence emotion regulation efforts and mental health, so the assessment of these beliefs is important. However, few psychometric measures are available, particularly for assessing the usefulness dimension. In this study (*N* = 161), we address this issue by developing and conducting an initial validation of a 16-item self-report measure called the Emotion Beliefs Questionnaire (EBQ). Confirmatory factor analyses found its structure to consist of three first-order factors: a controllability factor spanning both negatively and positively valenced emotions (*General-Controllability*), and two valence-specific usefulness factors (*Negative-Usefulness*, *Positive-Usefulness*). All first-order factors also loaded together on a higher-order factor, representing an overall maladaptive beliefs about emotions construct. All subscale and composite scores had good levels of internal consistency. Correlational and regression analyses found that EBQ scores related in expected ways with other measures, and were significant predictors of emotion regulation abilities and psychopathology. We conclude that the beliefs about emotions construct is multidimensional, and the EBQ appears to be a promising new tool to assess it.

Emotions are multidimensional phenomena, manifesting as responses across experiential, behavioral, and physiological channels of the emotion system [1]. They can be negatively or positively valenced, like sadness or happiness, and occur when people appraise a stimulus as being meaningful for their goals [2]. Research has shown that the beliefs people hold about mental phenomena such as emotions are important because they influence their responses to domain-relevant challenges and opportunities. People who believe, for example, that intelligence cannot be modified (i.e., entity theorists) are less likely to engage in efforts to develop their own cognitive abilities, as compared to people who think intelligence can change (i.e., incremental theorists) [see 3–4]. For beliefs about emotions, a similar pattern is evident. We now know that people differ, for example, in the extent to which they believe emotions are controllable. Tamir et al. [5] and others [e.g., 6] have shown that beliefs that emotions are uncontrollable are associated with lower emotion regulation self-efficacy, less usage of adaptive regulation strategies like cognitive reappraisal, poorer social adjustment, and more severe mental health symptoms.

Because beliefs about emotions appear to be an important correlate of long-term socioemotional outcomes [e.g., 5] their assessment is of interest to researchers and clinicians. However, few psychometric measures are available to assess beliefs about emotions, and as we argue below, existing tools have some conceptual limitations that may restrict their clinical and research utility. This is likely due, at least in part, to the fact that until recently few theoretical frameworks were available to organise different types of beliefs about emotions, or to conceptualise their impact on other important mental health variables like emotion regulation. Ford and Gross [7,8], however, recently introduced such a framework, and we think this added conceptual clarity presents an excellent opportunity to now develop more differentiated measures of beliefs about emotions. Our aim in this paper was therefore to try to address the existing measurement gaps, by introducing and validating the Emotion Beliefs Questionnaire (EBQ) as a new self-report tool for researchers and clinicians who want to work within the parameters of Ford and Gross’s [7,8] framework. Additionally, we sought to use this tool to further explore and understand the latent structure of the beliefs about emotions construct.

Prior to introducing the EBQ, we first describe the theoretical framework upon which we based our measure, then we outline a set of criteria from this framework that we think beliefs measures should ideally meet, and we review the content of existing measures against these criteria.

## Theoretical framework

Ford and Gross’s [7,8] framework for mapping beliefs about emotions focuses on two types of fundamental beliefs: beliefs about the *controllability* of emotions (i.e., the extent to which emotions are phenomena that can be modified and changed at will, or are phenomena that come and go as they please), and beliefs about the *usefulness* of emotions (i.e., the extent to which emotions are good vs bad, useful vs useless, valuable vs unimportant, helpful vs harmful, or desirable vs undesirable). While these are not the only types of beliefs about emotions people can hold, Ford and Gross [7,8] argue that these two are an important area of focus for the field, because these two sets of beliefs are conceptually separable from each other, are foundational in corresponding to longstanding philosophical debates about the nature of emotions [e.g., 9,10], and theoretically speaking, have important consequences for people’s emotion regulation skills.

Within this framework, these two types of beliefs can exist at different levels of specificity or abstraction. At the broadest level, which Ford and Gross [7,8] call superordinate beliefs, people have beliefs about the extent to which emotions, as a general construct, are controllable or useful. Subsumed within these superordinate beliefs, though, people might hold different beliefs about the controllability or usefulness of emotions depending on the specific properties of the emotions, situations, or targets in question (i.e., subordinate beliefs). These sets of subordinate beliefs include: (a) beliefs about specific *types of emotions*; (b) specific *emotion channels*, such as believing the behavioral manifestations of emotions are controllable, but the physiological and experiential channels are not; (c) specific *contexts*, such as believing that emotions are undesirable in the workplace, but important at home; and (d) specific *targets*, such as believing that emotions are controllable for adults but not for children, or beliefs about emotions for specific people (e.g., the self, a partner, a friend, etc.).

As noted by Ford and Gross [7,8], these beliefs about the controllability and usefulness of emotions are important, because they are likely to impact people’s efforts and performance at all stages of the emotion regulation process [2,11]. Conceptually, people who believe that emotions are uncontrollable should be less likely to try to regulate these emotions in the first place (i.e., because they doubt such regulation is possible), less likely to pick effective regulation strategies, and more likely to prematurely stop any ongoing regulation efforts. Similarly, people who believe emotions are useless may excessively try to down-regulate and get rid of (or not up-regulate) emotions, and be more likely to select and persist with regulation efforts they think will be effective for these goals [7,8]. Although empirical work in the emotion beliefs area is presently limited, available evidence does appear to support these types of predictions. For example, people who believe that emotions are generally uncontrollable report using adaptive emotion regulation strategies less frequently [e.g., 5] and are less motivated to engage in regulatory efforts to improve their well-being [12]. Similarly, experimental work has supported the idea that people are more likely to engage in regulatory efforts to experience emotions that they consider useful and avoid emotions they consider undesirable [e.g., 13,14]. Rigid beliefs in this usefulness domain also appear to be maladaptive in terms of their links with psychopathology, with therapy programs for borderline personality disorder often directly targeting patients’ beliefs that emotions are bad or stupid [15].

## Measuring beliefs about emotions

Based on this theoretical framework, we think measures of beliefs about emotions should ideally meet at least *three* criteria. First, because both types of beliefs about emotions are theoretically separable and important, a measure should be able to assess *both domains* separately (criterion one), that is, it should have items that assess the controllability domain and items that assess the usefulness domain. Second, to adequately assess the breadth of the construct, a measure should be able to assess the controllability and usefulness domains at the *superordinate level* (criterion two), that is, like commonly used measures of incremental or entity beliefs about intelligence [e.g., 3], it should assess people’s beliefs about emotions as a general construct, rather than just assess people’s beliefs specific to their own emotions or self-efficacy. Third, a measure of beliefs about emotions should, when desired, also be able to provide some information about beliefs at the *subordinate level* (criterion three). We think this might be of particular importance for providing valence-specific information about negative and positive emotions. For example, newer psychometric measures of other emotional constructs, such as emotion regulation [16–18], alexithymia [19] and emotional reactivity [20,21], provide valence-specific information, and have demonstrated that emotional constructs can operate differently depending on the valence of the emotion in question [see also, 22]. For the beliefs about emotions construct, this may also be the case, particularly given that certain psychopathologies are characterised by valence-specific abnormalities [see 23].

Unfortunately, no existing psychometric measure appears to meet all three of these criteria (see Table 1). To the best of our knowledge, there are five existing psychometric tools (all self-report questionnaires) that were specifically designed to measure beliefs about emotions. These are the Implicit Theories of Emotions Scale (ITES; [5]), the Beliefs about Emotions Scale (BES; [24]), the Attitudes Toward Emotions Scale (ATE; [25]), the Parents’ Beliefs about Children’s Emotions Questionnaire (PBACE; [26]), and the Emotion and Regulation Beliefs Scale (ERBS; [27]). It bears noting that some might also include in this list the large number of self-report measures of *emotion regulation ability or self-efficacy* (such as the Difficulties in Emotion Regulation Scale [DERS; 28] and the Perth Emotion Regulation Competency Inventory [PERCI; 17]); because due to their self-report nature these questionnaires are technically asking about people’s *beliefs* about whether they are able to control their own emotions (e.g., “When I’m feeling bad, I’m powerless to change how I’m feeling”). However, similar to the implicit beliefs tradition in the intelligence literature [3], we consider it conceptually useful to distinguish between those measures designed to assess implicit *beliefs about emotions in general* (e.g., “No matter how hard they try, people can’t really change the emotions that they have”) and measures designed to assess people’s own *emotion regulation ability or self-efficacy* (e.g., “When I’m feeling bad, I don’t know what to do to feel better”). In particular, we think maintaining a distinction between these constructs is important because of contemporary frameworks’ specifications that people’s beliefs about emotions impact on their own emotion regulation abilities [7,8]. With this in mind, below, we briefly describe each of the five existing measures designed to assess implicit beliefs about emotions.

**Table 1:** Existing psychometric measures of beliefs about emotions.

Measure	Authors and year of publication	Number of items	Meets the three conceptual criteria?		
			Criterion 1 (assesses both controllability and usefulness domains)	Criterion 2 (assesses domains at the superordinate level)	Criterion 3 (provides valence-specific scores)
Implicit Theories of Emotions Scale (ITES)	Tamir et al. (2007)[5]	4	✗	✓	✗
Beliefs about Emotions Scale (BES)	Rimes and Chalder (2010)[24]	12	✗	✗	✗
Attitudes Toward Emotions Scale (ATE)	Harmon-Jones et al. (2011)[25]	28	✗	✗	✓
Parents’ Beliefs about Children’s Emotions Questionnaire (PBACE)	Halberstadt et al. (2013)[26]	33	✓	✗	Partial
Emotion and Regulation Beliefs Scale (ERBS)	Veilleux et al. (2015)[27]	21	✗	✓	✗

### Implicit Theories of Emotions Scale

The ITES [5] was the first measure of beliefs about emotions developed, and is presently the most widely used tool in this area (e.g., [6, 13]). It is a 4-item self-report measure of the extent to which people think that emotions are controllable (e.g., “Everyone can learn to control their emotions”). All items are answered on a 6-point Likert scale, with higher ITES total scale scores indicating stronger beliefs that emotions are controllable. The ITES meets criterion two (i.e., measurement at superordinate level), as the items ask about beliefs about emotions in general. It does, however, not meet criterion one (i.e., assessing both belief domains) or criterion three (i.e., valence-specific measurement), because its items only target controllability, not usefulness, and none of its items specify a negative or positive valence.

### Beliefs about Emotions Scale

The BES [24] is a 12-item self-report measure of people’s beliefs about how acceptable it is for *them* to experience and express emotions (e.g., “It is a sign of weakness if I have miserable thoughts). Items are answered on a 7-point Likert scale, with higher scores indicating stronger beliefs that it is unacceptable for that respondent to experience and express emotions. In the standard scoring, all items are summed into a total scale score [24]. The BES does not meet criterion one because it does not provide separate subscale scores for the controllability and usefulness domains; in terms of content, in our view, many of the BES items appear to assess both controllability and usefulness (e.g., “If I lose control of my emotions in front of others, they will think less of me”). The BES also does not meet criterion two because its items are specific to beliefs about one’s own emotions, rather than people’s emotions in general, and it does not meet criterion three because no items ask about positive emotions.

### Attitudes Toward Emotions Scale

The ATE [25] is a 28-item measure of people’s beliefs about whether their emotions are desirable (i.e., useful). Items are answered on a 5-point Likert scale, with higher scores indicating stronger beliefs that an emotion is desirable. Separate subscale scores are derived for *Anger* (e.g., “I like the feeling of increased energy I get from expressing my anger”), *Joy* (e.g., I like experiencing joy), *Sadness* (e.g., I like it when movies make me feel sad, the sadder the better), *Disgust* (e.g., “If I hear something disgusting, I will listen to it again on purpose), and *Fear* (e.g., “I like to do things that scare me”). The ATE meets criteria two, because it has separate subscales for negative and positively valenced emotions. It does, however, not meet criterion one or criterion three. Its items only target usefulness, not controllability, and all its items are specific to beliefs about one’s own emotions, rather than people’s emotions in general.

### Parents’ Beliefs about Children’s Emotions Questionnaire

The PBACE [26] is a 33-item self-report measure of people’s beliefs about children’s emotions. Items are answered on a 6-point Likert scale, with seven subscale scores derived in the standard scoring: *Cost of positivity* (e.g., “Children may not focus on their commitments if they feel too much happiness”), *Value of anger* (e.g., “It is useful for children to feel angry sometimes”), *Manipulation* (e.g., “Children use emotions to manipulate others”), *Control* (e.g., “Children can control their emotions”), *Parental knowledge* (e.g., “Parents should encourage their child to tell them everything they are feeling”), *Autonomy* (e.g., “When children are sad, they need to find their own ways to move on”), and *Stability* (e.g., “Children’s emotions tend to be long-lasting”). The PBACE meets criterion one, because its *Control* subscale appears to measure the controllability dimension, and its *Cost of positivity* and *Value of anger* subscales appear to assess the usefulness dimension. It also partially meets criterion three, because these usefulness subscales have valence-specific items, though the *Control* subscale does not. The PBACE, however, does not meet criterion two, because it does not assess people’s beliefs about emotions in general (i.e., at the superordinate level); instead, its items are specific to beliefs about the emotions of children.

### Emotion and Regulation Beliefs Scale

The ERBS [27] is a 21-item self-report measure of beliefs that emotions can hijack self-control (*Hijack*; e.g., “When strong emotions are present, they dictate what a person says or does”), that emotion regulation is a worthwhile pursuit (*Regulation worth*; e.g., “Learning how to alter strong emotions is a worthwhile pursuit”), and that emotions constrain behaviour (*Emotional constraint*; e.g., “When people acknowledge their emotions, the emotions will completely take them over”). Separate subscale scores are derived for each of these categories. Items are answered on a 5-point Likert scale, with higher scores indicating stronger beliefs that emotions hijack self-control, that emotion regulation is worthwhile, or that emotions constrain behaviour. The ERBS meets criterion two, because all its items ask about people’s beliefs about emotions in general. It does, however, not meet criterion one, because none of its subscales clearly measure only the usefulness dimension. The *Emotional constraint* and *Hijack* subscales, for example, appear to have components of both controllability and usefulness in their items, in that these items are about emotions being uncontrollable and consequently leading to bad outcomes (e.g., “When a person feels really angry, it’s virtually impossible to not take the anger out on people or objects nearby”). The ERBS, additionally, does not meet criterion three, because it cannot provide any valence-specific scores; three items do specify a valence, but they exclusively refer to negative emotions, so no information about positive emotions can be derived from the ERBS.

## The present study

To address these limitations of the existing measures and provide a measure for those who want to work within the parameters of Ford and Gross’s [7,8] framework, we developed the EBQ. A copy of the measure and its scoring instructions are provided in the S2 File. The EBQ is a 16-item self-report measure of beliefs about emotions. It was designed to assess the controllability and usefulness dimensions delineated by Ford and Gross [7,8], and do so across negative and positive emotions. All items are answered on a 7-point Likert scale, with high scores indicating that respondents believe, in general, that emotions are uncontrollable and useless. Four valence-specific subscale scores were intended to be derived: *Negative-Controllability* (4 items; e.g., “Once people are experiencing negative emotions, there is nothing they can do about modifying them”), *Positive-Controllability* (4 items; e.g., “People cannot control their positive emotions”), *Negative-Usefulness* (4 items; e.g., “Negative emotions are harmful”), and *Positive-Usefulness* (e.g., “There is very little use for positive emotions”). Several theoretically meaningful composite scores were also intended to be derived, including summing the two controllability subscales into a *General-Controllability* composite and the two usefulness subscales into a *General-Usefulness* composite, to produce markers of people’s beliefs in these domains across both valence categories. As part of the development of the EBQ, we were also interested in exploring whether there might be statistical support for the summing of all 16 EBQ items into a total scale score; in other words, whether there would support for a higher-order beliefs about emotions factor, reflecting an overall marker of maladaptive beliefs about emotions. To the best of our knowledge, the potential presence of this type of higher-order factor has not been examined in any previous beliefs about emotions work.

We present the initial psychometric validation of the EBQ in this paper. We describe the item selection process, and examine the EBQ’s factor structure, internal consistency reliability, and concurrent/criterion validity. In terms of concurrent validity, we examined correlations between the EBQ and three other self-report measures of beliefs about emotions (ITES, ERBS, BES), as well as correlations with a marker of emotion regulation ability/self-efficacy and a marker of psychopathology symptoms.

## Method

### Participants

Ethics approval for this project was granted by the University of Western Australia Human Research Ethics Committee. All participants provided informed consent for their data to be used. Our sample was comprised of 161 adults (52.2% female) recruited from the general Australian population by an online survey recruitment company (Qualtrics panels). Participants were selected based on their age, gender, and geographic state, so as to get a sample with demographics reasonably representative of the adult population in Australia. Beyond these 161 adults, 36 additional participants also completed the survey, but their data were excluded in quality screening because they failed an attention check question (which asked them to select a specific point on a Likert scale) or they completed a questionnaire impossibly quickly (i.e., at a rate of less than 2 seconds per question), indicating inattentive responding. In the final sample of 161, participants’ average age was 47.03 years (*SD* = 18.02, range = 18–83) and 31.7% had a university degree as their highest level of completed education. Most (90.1%) were not currently university students. In terms of cultural background, 82% reported they were white/Caucasian and 13.7% reported they were Asian.

### Procedure

All 161 participants completed the EBQ as part of a battery of questionnaires in an online anonymous survey. We administered the EBQ in this study in an over-inclusive 30-item development form, with the intention of selecting the best subset of these items for retention in the final scale. We wrote these 30 development pool items to assess different aspects of the two superordinate beliefs about emotions (controllability and usefulness) proposed in Ford and Gross’s [7,8] framework. Half the items asked about beliefs about negative emotions, and the other half asked about beliefs about positive emotions (see S2 File for a list of all development pool items). The writing of these 30 development pool items went through two stages. First, we wrote an initial draft of the items, and then they were checked for readability and comprehension by two psychiatrists, two clinical psychologists, and three high school teachers. Then, based on feedback from these professionals, we edited some items’ phrasing, and this formed the final set of 30 items that were administered to participants in this study.

Based on some preliminary exploratory and confirmatory factor analyses of our sample of 161 participants’ responses on these 30 items, we selected the best 16 items to form the final measure (the results of these preliminary analyses are not reported in this paper, but some are provided in the S2 File). Item selection was based on two main criteria. First, in terms of content validity, to properly capture the breadth of the construct we wanted an even number of items in each of the hypothesised four subscales. Subscale size was set at four items, because we thought this provided a good balance between brevity, content comprehensiveness, and statistical reliability (e.g., most authors recommend that subscales have at least 3 items, with more items likely to increase reliability [29]). Second, when subjected to factor analyses, we required that all retained items load well (i.e., factor loadings ≥ .40; [30]) on a theoretically congruent latent factor, and not cross-load over multiple factors. Although a pool of 30 EBQ items were administered to our sample of 161 adults, in this paper we report the results of analyses that include *only* the 16 items we decided to retain in the final scale.

### Materials

In addition to the EBQ items, the survey battery also included the Implicit Theories of Emotions Scale (ITES), the Emotion and Regulation Beliefs Scale (ERBS), the Beliefs about Emotions Scale (BES), the Perth Emotion Regulation Competency Inventory (PERCI), and the Depression Anxiety Stress Scales-21 (DASS-21). The ITES, ERBS and BES were described earlier, so we detail the PERCI and DASS-21 below.

The PERCI [17] is a 32-item self-report measure of people’s ability to regulate their own negative and positive emotions. Items are answered on a 7-point Likert scale, with higher scores indicating more emotion regulation difficulties. Several subscale and composite scores can be derived, and we focus only on the composite scores in this study. These include a *Negative-Emotion regulation* composite (e.g., “When I’m feeling bad, I don’t know what to do to feel better”) and a *Positive-Emotion regulation* composite (e.g., “When I’m feeling good, I have no control over whether that feeling stays or goes”), which can be used as overall markers of difficulty regulating emotions for negative or positive emotions, respectively. All items can also be summed into a *General-Emotion regulation* composite, as an overall index of emotion regulation ability across both valence domains. The PERCI has demonstrated good validity and reliability [e.g., 17].

The DASS-21 [31] is a 21-item self-report measure of depression, anxiety, and stress symptoms experienced in the past week. Separate subscale scores can be derived for each symptom category, and all items can also be summed into a total scale score as an overall marker of psychological distress. Items are answered on a 4-point Likert scale, with higher scores indicating more severe symptoms. The DASS-21 has demonstrated good validity and reliability [e.g., 31].

### Analytic strategy

AMOS 25 software was used for confirmatory factor analyses (CFAs) and SPSS 25 was used for all other analyses. The 16 EBQ items used in our analyses were reasonably normally distributed (average skewness = .68, average kurtosis = .53).

#### Factor structure

**Fig 1:**
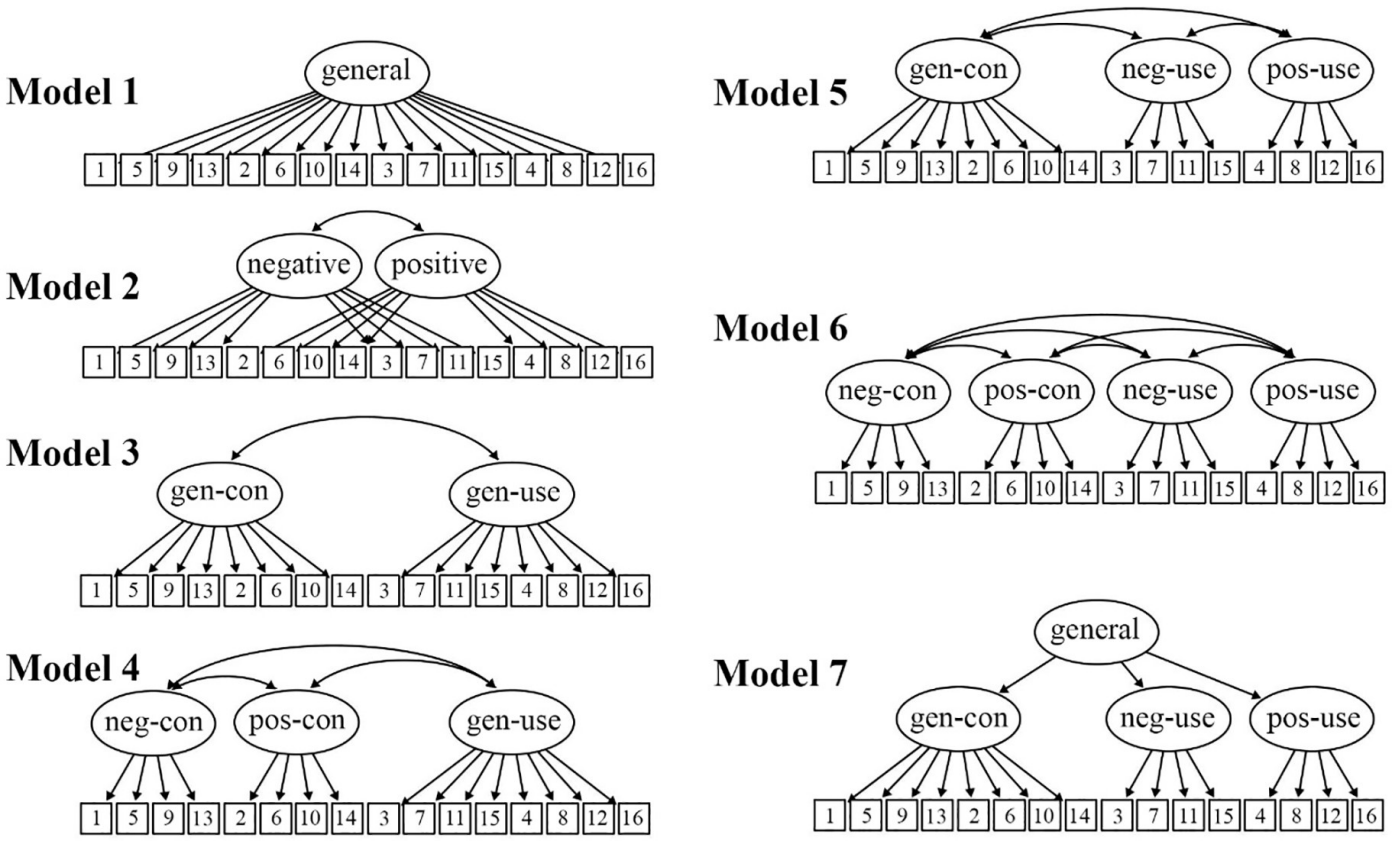
A visual representation of the different confirmatory factor analysis models tested for the 16-item EBQ. Squares represent items and ellipses represent latent factors. All items had an associated error term. Item numbering here reflects the order we recommend items be administered in for the 16-item EBQ. Gen-con = General-Controllability, gen-use = General-Usefulness, neg-con = Negative-Controllability, pos-con = Positive-Controllability, neg-use = Negative-Usefulness, pos-use = Positive-Usefulness.

We conducted a series of CFAs (maximum likelihood estimation based on a Pearson covariance matrix) to examine the factorial validity of the 16-item EBQ and the latent structure of the construct. We used CFA for these main analyses because it is considered appropriate for hypothesis testing when there is a theoretical rationale to expect a certain factor structure [e.g., 32–34]. Most authors recommend that at least 100 participants or five participants per variable in the analysis are required for robust factor analysis [e.g., 35,36], so we considered our sample size of 161 to be sufficient here. We examined several theoretically informed models of increasing complexity (see Fig 1).

First, we examined six first-order models to determine which factors best represented the first-order factor structure of the measure. Model 1 was a 1-factor model, where all 16 items were specified to load on a single factor. Model 2 was a 2-factor correlated model, where items were separated based on valence, but no distinction was made between the controllability or usefulness components; items were specified to load on correlated “*Negative valence*” or “*Positive valence*” factors. Model 3 was also a 2-factor correlated model, where a distinction was made between the controllability and usefulness components, but no distinction was made based on valence; items were specified to load on correlated “*General-Controllability*” or “*General-Usefulness*” factors. Model 4 was a 3-factor model, where a distinction was made between the controllability and usefulness components, but a valence distinction was made only for the controllability component; items were specified to load on correlated “*Negative-Controllability*”, “*Positive-Controllability*”, and “*General-Usefulness*” factors. Model 5 was a 3-factor model, where a distinction was made between the controllability and usefulness components, but a valence distinction was made only for the usefulness component; items were specified to load on correlated “*General-Controllability*”, “*Negative-Usefulness*”, and “*Positive-Usefulness*” factors. Model 6 was a 4-factor model, where a distinction was made between the controllability and usefulness components, and a valence distinction was made for both components; items were specified to load on correlated “*Negative-Controllability*”, “*Positive-Controllability*”, “*Negative-Usefulness*”, and “*Positive-Usefulness*” factors.

Following this, in Model 7, we examined a higher-order version of the best fitting first-order model; the first-order factors were specified to load together on a higher-order general factor. Our aim here was to establish whether the superordinate controllability and usefulness domains delineated by Ford and Gross [7,8] could form parts of a coherent, multidimensional, beliefs about emotions construct.

Model goodness-of-fit was judged used three fit indexes: the comparative fit index (CFI), Tucker-Lewis index (TLI), and root mean square error of approximation (RMSEA). CFI and TLI values ≥ .90 were judged to indicate acceptable fit, as were RMSEA values ≤ .08 [37]. The Akaike information criterion (AIC) was also used to directly compare the fit of the models; AIC penalises model complexity and lower values indicate a better fitting model [38]. Factor loadings ≥ .40 were considered meaningful loadings [30].

#### Descriptive statistics and internal consistency reliability

Descriptive statistics and Cronbach’s alpha internal consistency reliability coefficients were reported for all EBQ subscale and composite scores. To examine potential gender and age differences, we conducted a series of ANCOVAs using EBQ scores as the dependent variables, participant gender as the independent variable, and participant age as a covariate.

#### Concurrent and criterion validity

*Pearson correlations*. Pearson correlations were calculated between EBQ scores and ITES, ERBS, BES, DASS-21, and PERCI scores. We expected that EBQ scores would correlate with scores from the other beliefs about emotions measures, particularly those scores that were designed to assess the same or similar constructs (e.g., the ITES total scale score and the EBQ *General-Controllability* composite). Similarly, because of the hypothesised links between emotion regulation abilities and beliefs about emotions, we expected that high EBQ scores would be associated with more difficulties regulating one’s own emotions, and higher levels of depression, anxiety, and stress symptoms.

*Regression analyses*. We also conducted a set of multiple regression analyses, to examine whether EBQ scores could predict significant variance in psychopathology symptoms and emotion regulation abilities. First, we conducted several separate multiple regression analyses, where all the scores reflecting a supported first-order factor (i.e., subscale) of the EBQ were entered as predictor variables, and the criterion variable of interest was either the DASS-21 *Depression*, *Anxiety*, or *Stress* scores, or the PERCI *Negative-Emotion regulation* or *Positive-Emotion regulation* scores.

Next, we conducted another set of multiple regression analyses (still focused on the same criterion variables) to examine more closely whether the EBQ provided additional predictive value beyond that of the ITES, and whether measuring the usefulness component added value beyond measuring just the controllability component. We therefore used a stepped entry procedure here. First, we entered the ITES total scale score into the regression model, then we added the EBQ *General-Controllability* score, and lastly we added the EBQ *Negative-Usefulness* and *Positive-Usefulness* scores. The ITES was selected as the comparison point for the EBQ because, as aforementioned, it is presently the most widely used measure of beliefs about emotions [e.g., 13], and conceptually it is the existing measure that most cleanly aligns with a superordinate belief component (i.e., controllability) from Ford and Gross’s [7,8] framework.

## Results

### Factor structure

Our confirmatory factor analyses found that, overall, Model 5 (3-factor correlated model) was the best solution and a good fit to the data. Goodness-of-fit index values, factor loadings, and factor intercorrelations are displayed in Tables 2, 3 and 4, respectively.

**Table 2:** Goodness-of-fit index values for the different confirmatory factor analysis models of the 16-item EBQ.

Model	Factors	Χ^2^ (*df*)	CFI	TLI	RMSEA (90% CI)	AIC
1	1-factor	477.647 (104)	.689	.642	.150 (.136-.164)	541.647
2	2-factors	475.222 (103)	.691	.640	.150 (.137-.164)	541.222
3	2-factors	391.428 (103)	.760	.721	.132 (.119-.146)	457.428
4	3-factors	388.622 (101)	.761	.716	.133 (.120-.148)	458.622
5	3-factors	188.661 (101)	.927	.913	.074 (.057-.090)	258.661
6	4-factors	177.129 (98)	.934	.919	.071 (.054-.088)	253.129
7	Higher-order model	196.648 (102)	.921	.907	.076 (.060-.092)	264.648

For all models, Χ^2^ *p* < .05. CFI = comparative fit index, TLI = Tucker-Lewis index, RMSEA = root mean square error of approximation, AIC = Akaike information criterion, CI = confidence interval.

**Table 3:** Standardised factor loadings from confirmatory factor analyses of the 16 retained EBQ items (for Model 5 and Model 7).

Item/factor	3-factor model (Model 5)	Higher-order model (Model 7)
**General-Controllability**		.85^a^
1—Once people are experiencing negative emotions, there is nothing they can do about modifying them.	.82	.82
5—It doesn’t matter how hard people try, they cannot change their negative emotions.	.73	.72
9—People cannot control their negative emotions.	.58	.51
13—People cannot learn techniques to effectively control their negative emotions.	.91	.91
2—People cannot control their positive emotions.	.45	.44
6—People cannot learn techniques to effectively control their positive emotions.	.71	.70
10—It doesn’t matter how hard people try, they cannot change their positive emotions.	.69	.69
14—Once people are experiencing positive emotions, there is nothing they can do about modifying them.	.71	.70
**Negative-Usefulness**		.46^a^
3—There is very little use for negative emotions.	.68	.70
7—People don’t need their negative emotions.	.77	.81
11—Negative emotions are harmful.	.76	.77
15—The presence of negative emotions is a bad thing for people.	.69	.70
**Positive-Usefulness**		.79^a^
4—Positive emotions are very unhelpful to people.	.75	.75
8—There is very little use for positive emotions.	.86	.85
12—People don’t need their positive emotions.	.71	.71
16—Positive emotions are harmful.	.62	.63

^a^Loading of first-order factor on higher-order factor. All factor loadings were statistically significant, *p* < .001. Item numbering here reflects the order we suggest the items be presented in for the 16-item EBQ. For a list of the order the items were administered in within the larger 30-item development pool, see the S2 File.

**Table 4:** Estimated factor intercorrelations from confirmatory factor analyses of the 16-item EBQ.

Model/factor	Factor		
	F1	F2	F3
**Model 2**			
F1 Negative valence	-	-	-
F2 Positive valence	.96**	-	-
**Model 3**			
F1 General-Controllability	-	-	-
F2 General-Usefulness	.72*	-	-
**Model 4**			
F1 Negative-Controllability	-	-	-
F2 Positive-Controllability	1.05**	-	-
F3 General-Usefulness	.72*	.75*	-
**Model 5**			
F1 General-Controllability	-	-	-
F2 Negative-Usefulness	.31**	-	-
F3 Positive-Usefulness	.70**	.18	-
**Model 6**			
F1 Negative-Controllability	-	-	-
F2 Positive-Controllability	1.05**	-	-
F3 Negative-Usefulness	.27**	.43**	-
F4 Positive-Usefulness	.70**	.71**	.18

**p < .01,

*p < .05

The 1-factor model (Model 1) was a poor fit to the data, highlighting that the 16-item EBQ was measuring a multidimensional construct. Model 2 did not improve levels of fit, indicating that it was insufficient to just make a distinction between positive and negative valence. Model 3, which distinguished between the controllability and usefulness components of beliefs about emotions (but did not distinguish between positive and negative valence), improved fit slightly, but was still a poor fit according to all examined fit indexes. Model 4 (and Model 6), in turn, highlighted that it was unnecessary to distinguish between negative and positive valence for the controllability component, as the “*Negative-Controllability*” and “*Positive-Controllability*” factors in this model were extremely highly correlated (estimated *r* = 1.05, *p* < .001), with the correlation over 1.0 indicating model problems. Conversely, Model 5 highlighted that it was important to distinguish between negative and positive valence for the usefulness component, as the “*Negative-Usefulness*” and “*Positive-Usefulness*” factors were not significantly correlated with each other (estimated *r* = .18, *p* = .068). These “*Negative-Usefulness*” and “*Positive-Usefulness*” factors were both significantly correlated with the “*General-Controllability*” factor (estimated *r*s = .31-.70, *p*s < .01). Model 5 displayed good levels of fit according to CFI, TLI, and RMSEA, with all items loading well on their intended factor (factor loadings = .45-.91). The first-order factor structure of the EBQ therefore appeared to be well represented in CFA by a single controllability factor spanning both valence categories (“*General-Controllability*”) and two valence-specific usefulness factors (“*Negative-Usefulness*” and “*Positive-Usefulness*”). It should be noted that if an unrestricted exploratory factor analysis is conducted on the EBQ items, this same 3-factor structure emerges (accounting for 62.38% of the variance in item scores; see S1 Table in the supplementary materials).

In our higher-order CFA model (Model 7), we tested whether these three first-order factors from Model 5 could all load well together on a higher-order general factor, and we found that they could (factor loadings = .46-.85). The higher-order model had slightly lower fit index values than its equivalent correlated model (indicating that the general factor did not perfectly account for the relationship between the three first-order factors), but it nonetheless maintained acceptable levels of fit according to all examined fit indexes. We therefore judged a higher-order beliefs about emotions factor to be tenable.

### Descriptive statistics and internal consistency reliability

Descriptive statistics and Cronbach’s alpha reliability coefficients for the EBQ subscale and composite scores are provided in Table 5. Although our factor analytic results suggest that the *General-Controllability* composite score should ideally be used in place of the separate *Negative-Controllability* and *Positive-Controllability* subscale scores (as these two valence-specific subscales appear to measure the same first-order factor), we nonetheless report values for these two subscales in the interest of completeness. Similarly, because our factor analysis supported the tenability of a higher-order factor, we report values for an EBQ total scale score comprised of all 16 items. This total scale score represents a composite of the extent to which participants think emotions are uncontrollable and useless across both valence categories, and therefore appears to be an overall index of maladaptive beliefs about emotions. All EBQ subscale and composite scores had acceptable to good levels of internal consistency reliability (α = .70-.88).

**Table 5:** Descriptive statistics and Cronbach’s alpha reliability coefficients for the EBQ, ITES, BES, ERBS, DASS-21, and PERCI.

	Total sample (*N* = 161)				Males (*N* = 77)		Females (*N* = 84)	
	*M*	*SD*	*range*	*α*	*M*	*SD*	*M*	*SD*
**EBQ**								
***Subscales***								
Negative-Controllability	11.63	4.62	4–28	.84	11.91	4.64	11.38	4.62
Positive-Controllability	12.26	3.92	4–28	.70	12.88	3.73	11.69	4.02
Negative-Usefulness	15.41	5.37	4–28	.82	15.34	5.38	15.48	5.39
Positive-Usefulness	8.24	4.39	4–28	.83	9.32	5.04	7.25	3.43
***Composites***								
General-Controllability	23.89	8.13	8–56	.88	24.79	8.03	23.07	8.19
General-Usefulness	23.65	7.47	8–56	.77	24.66	8.09	22.73	6.77
Total scale	47.55	13.69	17–112	.88	49.46	14.45	45.80	12.78
**ITES**								
Total scale	14.55	3.49	6–22	.80	15.05	3.40	14.08	3.53
**BES**								
Total scale	34.11	12.71	8–72	.90	35.31	12.13	33.01	13.20
**ERBS**								
Emotional constraint	25.40	7.27	9–45	.87	25.91	7.46	24.94	7.10
Regulation worth	27.81	3.62	18–35	.79	27.81	3.48	27.81	3.77
Hijack	17.62	3.23	9–25	.70	17.71	3.47	17.54	3.02
**DASS-21**								
Depression	4.85	5.20	0–21	.94	4.52	5.12	5.14	5.29
Anxiety	3.55	4.04	0–21	.86	2.95	4.01	4.10	4.01
Stress	5.30	4.20	0–21	.86	4.62	4.05	5.93	4.26
Total scale	13.70	12.24	0–63	.95	12.09	12.03	15.17	12.33
**PERCI**								
Negative-Emotion Regulation	58.47	17.90	16–112	.92	58.43	16.49	58.51	19.20
Positive-Emotion Regulation	36.86	16.11	16–112	.94	39.90	17.01	34.08	14.80
General-Emotion Regulation	95.34	30.22	32–224	.95	98.33	30.17	92.60	30.19

EBQ = Emotion Beliefs Questionnaire, ITES = Implicit Theories of Emotions Scale, BES = Beliefs about Emotions Scale, ERBS = Emotion and Regulation Beliefs Scale, DASS-21 = Depression Anxiety Stress Scales-21, PERCI = Perth Emotion Regulation Competency Inventory.

An ANCOVA comparing EBQ total scale scores between genders indicated that, overall, males reported more maladaptive beliefs about emotions than females, *F*(1, 158) = 4.107, *p* = .044, partial ƞ^2^ = .025. At the subscale level, there were no gender differences for the EBQ *General-Controllability* or *Negative-Usefulness* scores (*p*s > .05), but compared to females’ beliefs, males believed positive emotions were more useless, *F*(1, 158) = 10.487, *p* = .001, partial ƞ^2^ = .062. Age was not a significant covariate in any of these analyses (*p*s > .05), suggesting that these adults’ beliefs about emotions did not differ based on age. In terms of the distinction between *Negative-Usefulness* and *Positive-Usefulness* scores, for the total sample, a paired samples *t*-test highlighted that participants generally considered positive emotions to be significantly more useful than negative emotions, *t*(160) = 14.317, *p* < .001, Cohen’s *d* = .21.

### Concurrent validity

Correlations between the administered measures for the total sample are displayed in Table 6. EBQ scores correlated significantly (*p* < .05) in expected ways with various scores from other measures of beliefs about emotions. The EBQ *General-Controllability* composite was significantly negatively correlated with the ITES total scale score (*r* = -.45), whereas the EBQ *Negative-Usefulness* (*r* = .04) and *Positive-Usefulness* subscales (*r* = -.04) were uncorrelated with the ITES. These results highlight that while the controllability items of the EBQ assess a similar construct to the ITES, the EBQ usefulness items assess aspects of beliefs about emotions not captured by the ITES. For the ERBS, all EBQ subscale and composite scores were significantly positively correlated with the ERBS *Emotional constraint* subscale (*r*s = .36 to .63), but only those EBQ scores focused on controllability were correlated (negatively, as expected) with the ERBS *Regulation worth* subscale (*r*s = .06 to -.24). Similarly, the ERBS *Hijack* subscale score was significantly positively correlated with all EBQ subscale scores, except for *Positive-Usefulness* (*r*s = .09 to .33). The BES total scale score was significantly positively correlated with all EBQ subscales and composite scores (*r*s = .18 to .40). These findings are therefore consistent with our suggestions that many of the ERBS and BES subscale scores are not pure markers of controllability or usefulness, but rather appear to assess elements of both these domains.

**Table 6:** Pearson correlations between EBQ scores and ITES, BES, ERBS, DASS-21, and PERCI scores.

	EBQ						
	Subscales				Composites		
	Negative-Controllability	Positive-Controllability	Negative-Usefulness	Positive-Usefulness	General-Controllability	General-Usefulness	Total scale
**ITES**							
Total scale	-.48**	-.37**	.04	-.04	-.45**	.00	-.27**
**BES**							
Total scale	.23**	.37**	.40**	.18*	.31**	.39**	.40**
**ERBS**							
Emotional constraint	.48**	.56**	.49**	.36**	.54**	.56**	.63**
Regulation Worth	-.24**	-.17*	.15	-.09	-.22**	.06	-.10
Hijack	.27**	.29**	.33**	.09	.29**	.29**	.33**
**DASS-21**							
Depression	.19*	.20*	.16*	.15	.21**	.20*	.23**
Anxiety	.39**	.36**	.26**	.27**	.40**	.35**	.43**
Stress	.35**	.31**	.20*	.18*	.35**	.25**	.34**
Total scale	.33**	.31**	.22**	.21**	.34**	.28**	.36**
**PERCI**							
Negative-Emotion regulation	.50**	.53**	.33**	.14	.54**	.32**	.50**
Positive-Emotion regulation	.59**	.62**	.20*	.55**	.64**	.47**	.63**
General-Emotion Regulation	.61**	.65**	.31**	.38**	.66**	.44**	.63**

**p < .01,

*p < .05.

EBQ = Emotion Beliefs Questionnaire, ITES = Implicit Theories of Emotions Scale, BES = Beliefs about Emotions Scale, ERBS = Emotion and Regulation Beliefs Scale, DASS-21 = Depression Anxiety Stress Scales-21, PERCI = Perth Emotion Regulation Competency Inventory.

EBQ scores also correlated in expected ways with markers of psychopathology symptoms and emotion regulation ability or self-efficacy. All EBQ subscale and composite scores were significantly positively correlated with higher levels of depression, anxiety, and stress symptoms. Of all the EBQ scores, the total scale score had the highest correlations with depression (*r* = .23) and anxiety (*r* = .43), thus highlighting the potential clinical relevance of using this total scale score as an overall marker of maladaptive beliefs about emotions. All EBQ subscale and composite scores, similarly, were significantly positively correlated with most of the PERCI composite scores (*r*s = .14 to .66), thus reinforcing the hypothesised links between emotion regulation ability and people’s general beliefs about emotions. Some valence-specific relationships were evident here. The EBQ *Positive-Usefulness* subscale, for example, was significantly correlated with the ability to regulate positive emotions (*r* = .55) and uncorrelated with the ability to regulate negative emotions (*r* = .14) (difference between these correlations, *p* < .001).

### Criterion validity

Our multiple regression analyses reinforced that EBQ scores were significant predictors of psychopathology symptoms and emotion regulation abilities, with different EBQ scores being more important predictors depending on the construct of interest (for standardised beta coefficients, see Table 7). We used the *General-Controllability* score in these regression analyses, rather than the separate *Negative-Controllability* and *Positive-Controllability* scores, because the *General-Controllability* score was supported in our factor analyses. In these regression analyses using the EBQ *General-Controllability*, *Negative-Usefulness*, and *Positive-Usefulness* scores as the predictors, all three EBQ scores were significant (*p* < .05) predictors of people’s ability to regulate negative emotions (variance explained by the overall model = 35.6%), whereas only *General-Controllability* and *Positive-Usefulness* (not *Negative-Usefulness*) were significant predictors of people’s ability to regulate their positive emotions (variance explained by the overall model = 46.3%). For depression symptoms, none of the three EBQ scores were significant unique predictors (*p*s = .128-.578), though the variance accounted for by the overall model was still statistically significant (variance explained = 5.4%, *p* = .032). For anxiety symptoms, *General-Controllability* and *Negative-Usefulness* (not *Positive-Usefulness*) were significant predictors (variance explained by the overall model = 18.4%), and for stress symptoms only *General-Controllability* was a significant predictor (variance explained by the overall model = 13.2%).

**Table 7:** Standardised beta (β) coefficients from multiple regression analyses using EBQ scores as the predictor variables, and psychopathology symptoms (depression, anxiety, stress) or emotion regulation abilities (for negative or positive emotions) as the criterion variables.

Predictor variables	Criterion variable (standardised β coefficients)				
	Depression	Anxiety	Stress	Negative-Emotion regulation	Positive-Emotion regulation
EBQ General-Controllability	.15	.31*	.33*	.60*	.48*
EBQ Negative-Usefulness	.10	.16	.10	.19*	.01
EBQ Positive-Usefulness	.05	.08	-.02	-.22*	.28*

*Significant predictor within the regression model, *p* < .05.

We also found that these EBQ scores added substantial prediction value above that of the ITES. The ITES total scale score, by itself, predicted 0.0%, 0.0%, 1.7%, 6.2%, and 4.5% of the variance in depression, anxiety, stress, negative emotion regulation ability, and positive emotion regulation ability, respectively. Adding just the EBQ *General-Controllability* score into the model significantly improved the predictive strength of the model, accounting for an additional 4.5%, 15.9%, 9.9%, 22.2%, and 36.8% of the variance in depression, anxiety, stress, negative emotion regulation ability, and positive emotion regulation ability, respectively (all model *p*s < .05). Adding the EBQ *Negative-Usefulness* and *Positive-Usefulness* scores into the model, in turn, tended to improve prediction strength further, accounting for an additional 0.9%, 2.0%, 1.0%, 6.6%, 5.0% of the variance in depression, anxiety, stress, negative emotion regulation ability, and positive emotion regulation ability, respectively.

In sum, there was good support in these data for the clinical relevance of EBQ scores, as well as for the incremental utility of assessing beliefs about emotions in terms of both the controllability and usefulness components, and assessing across both valence domains.

## Discussion

Our aim here was to introduce the EBQ, conduct the initial validation study of its properties, and use this new measure to further explore the latent structure of the beliefs about emotions construct. Overall, the EBQ appeared to have good validity and reliability in our adult sample.

### Structure of emotion beliefs

The factor structure of the EBQ was well represented by three correlated first-order factors: a controllability factor spanning both valence domains (*General-Controllability*), and two valence-specific usefulness factors (*Negative-Usefulness*, *Positive-Usefulness*). There was, moreover, good evidence to support the presence of a higher-order factor, representing people’s overall level of maladaptive beliefs about emotions. These results allow us to make some key conclusions about the latent structure of the construct.

First, our results with the EBQ suggest that emotional valence is an important determinant of people’s beliefs about emotions, but only in terms of usefulness. In this sample, negative emotions were generally considered to be more useless than positive emotions, whereas negative emotions and positive emotions were considered to have similar levels of controllability. Second, our results highlight that beliefs about controllability and usefulness are statistically separable components of a coherent, multidimensional construct. These preliminary findings therefore support Ford and Gross’s [7,8] theoretical delineation of controllability and usefulness as two separable sets of beliefs about emotions, and also support their proposal that emotional valence is an important subordinate consideration. Our findings expand on this work by being the first to statistically test for a higher-order beliefs about emotions factor. The presence of this higher-order factor indicates that beliefs about controllability and usefulness do not operate independently of each other, at least statistically, but rather appear to be two linked parts of a broader network of maladaptive (or adaptive) beliefs about emotions. All EBQ subscale and composite scores had acceptable or good reliability, and so these scores appear to be able to robustly assess this multidimensional construct at different levels of specificity or abstraction.

### Clinical implications

The potential clinical relevance of the EBQ was further demonstrated in this study via its pattern of correlations with other established measures. For example, in line with contemporary theorising [7,8], stronger beliefs that emotions were uncontrollable and useless (i.e., high EBQ scores) were significantly associated with poorer emotion regulation abilities and higher levels of depression, anxiety, and stress symptoms.

One unexpected finding in this area that bears noting was that some EBQ scores were more highly correlated with emotion regulation abilities than they were with other measures of beliefs about emotions. Some might interpret this as a discriminant validity issue, but the content validity of all EBQ items does look sound to us, and conceptually these items are clearly distinguishable from the items of emotion regulation ability measures like the PERCI. We think these high correlations could, more likely, actually represent a strength of the EBQ, in terms of the EBQ potentially tapping the beliefs about emotions construct in a manner that is more comprehensive and clinically relevant than older beliefs measures. Indeed, in our sample, the EBQ was able to predict more variance than the ITES in people’s emotion regulation abilities and psychopathology symptoms. This extra predictive power appears to be partly due to the EBQ also assessing the usefulness dimension (instead of just controllability), but the EBQ *General-Controllability* score by itself also performed better than the ITES total scale in this respect. Such findings will require replication in other samples, but given that older beliefs measures like the ITES cannot assess both the controllability and usefulness dimensions, or do not account for valence, our available data suggest that the EBQ may represent a useful measurement advancement.

### Limitations and future directions

We think our introduction of the EBQ makes a strong contribution, but some limitations of our study should be noted that will require future research. First, we have proposed the EBQ as a 16-item measure here, but it was administered to our sample as part of a larger pool of items. Our selection of the best performing 16 items from this larger item pool will have optimised the scale around this sample, so it will be important for future work to test the replicability of our findings in other samples. Second, our sample size was modest, although as noted above, it was still large enough for robust factor analysis according to commonly used criteria [35,36]. Third, we did not test the performance of the EBQ in clinical or adolescent populations, so it is presently unclear whether the structure of the beliefs about emotions construct operates similarly in these population types. It is possible that in clinical samples, for instance, beliefs about the controllability of negative emotions and beliefs about the controllability of positive emotions might be more differentiated. This possibility is one reason why we maintained the ability to derive valence-specific controllability subscales in the EBQ (the other reason being to maximise functionality for valence-specific research questions). Future studies comparing the performance of the EBQ across different population types would therefore be useful, particularly with a view to further exploring the validity and robustness of the EBQ high-order factor score, and the similarities and differences between the EBQ and other emotion beliefs measures like the ITES. We also did not examine the test-retest reliability of the EBQ, so the extent to which beliefs about emotions are stable over time will be an important question for future research.

In sum, our data suggest that the beliefs about emotions construct is multidimensional, and that the EBQ is a promising new tool to assess it. Apparent strengths of the measure include its capacity to assess both the controllability and usefulness dimensions, and to do so for both negative and positive emotions. While more work is needed to confirm these findings in other sample types, we think use of the EBQ in future studies should help to enable a more comprehensive understanding of beliefs about emotions.

## Supporting information

S1 Table An ordered list of the 30 administered EBQ items in the development pool. *Item retained in the 16-item EBQ.
(DOCX)

S2 Table Factor loadings from an exploratory factor analysis of the 16 retained EBQ items. Factor loadings < .20 are not displayed. Principal axis factoring with direct oblimin rotation was used. Three factors were extracted (eigenvalues > 1) accounting for 62.38% of the variance in item scores. Correlations between the three factors were as follows: F1-F2 = .28, F1-F3 = .49, F2-F3 = .18.
(DOCX)

S1 File (SAV)

S2 File (PDF)

## References

[1] Mauss IB, Levenson RW, McCarter L, Wilhelm FH, Gross JJ. The tie that binds? Coherence among emotion experience, behavior, and physiology. Emotion. 2005 6;5(2):175 10.1037/1528-3542.5.2.175 15982083

[2] Gross JJ. Emotion regulation: Current status and future prospects. Psychol Inq. 2015 1 2;26(1):1–26.

[3] Dweck CS. Motivational processes affecting learning. Am psychol. 1986 10;41(10):1040.

[4] Hong YY, Chiu CY, Dweck CS, Lin DM, Wan W. Implicit theories, attributions, and coping: a meaning system approach. J Pers Soc Psychol. 1999 9;77(3):588.

[5] Tamir M, John OP, Srivastava S, Gross JJ. Implicit theories of emotion: Affective and social outcomes across a major life transition. J Personality Soc Psychol. 2007 4;92(4):731. 10.1037/0022-3514.92.4.731 17469955

[6] De Castella K, Goldin P, Jazaieri H, Ziv M, Dweck CS, Gross JJ. Beliefs about emotion: Links to emotion regulation, well-being, and psychological distress. Basic Appl Soc Psych. 2013 11 1;35(6):497–505.

[7] Ford BQ, Gross JJ. Emotion regulation: Why beliefs matter. Can Psychol. 2018 2;59(1):1.

[8] Ford BQ, Gross JJ. Why beliefs about emotion matter: An emotion-regulation perspective. Curr Dir Psychol Sci. 2019 2;28(1):74–81.

[9] Epictetus. The discourses of Epictetus: With the Encheiridion and fragments Translated, with notes, a life of Epictetus, and a view of his philosophy. London: G. Bell and Sons; 1906.

[10] Hume D. A Treatise of Human Nature. London: John Noon; 1739.

[11] Gross JJ. The emerging field of emotion regulation: An integrative review. Rev Gen Psychol. 1998 9;2(3):271–99.

[12] Howell AJ, Passmore HA, Holder MD. Implicit theories of well-being predict well-being and the endorsement of therapeutic lifestyle changes. J Happiness Stud. 2016 12 1;17(6):2347–63.

[13] Tamir M, Ford BQ. When feeling bad is expected to be good: Emotion regulation and outcome expectancies in social conflicts. Emotion. 2012 8;12(4):807 10.1037/a0024443 21728413

[14] Dennis PA, Halberstadt AG. Is believing seeing? The role of emotion-related beliefs in selective attention to affective cues. Cognition Emotion. 2013 1 1;27(1):3–20. 10.1080/02699931.2012.680578 22712535

[15] Linehan M. Cognitive-behavioral treatment of borderline personality disorder. New York: Guilford Press; 1993.

[16] Weiss NH, Gratz KL, Lavender JM. Factor structure and initial validation of a multidimensional measure of difficulties in the regulation of positive emotions: The DERS-Positive. Behav Modif. 2015 5;39(3):431–53. 10.1177/0145445514566504 25576185 PMC4420643

[17] Preece DA, Becerra R, Robinson K, Dandy J, Allan A. Measuring emotion regulation ability across negative and positive emotions: The Perth Emotion Regulation Competency Inventory (PERCI). Pers Indiv Differ. 2018 12 1;135:229–41.

[18] Zou C, Plaks JE, Peterson JB. Don’t get too excited: Assessing individual differences in the down-regulation of positive emotions. J Pers Assess. 2019 1 2;101(1):73–83. 10.1080/00223891.2017.1339711 28678548

[19] Preece D, Becerra R, Robinson K, Dandy J, Allan A. The psychometric assessment of alexithymia: Development and validation of the Perth Alexithymia Questionnaire. Pers Indiv Differ. 2018 10 1;132:32–44.

[20] Becerra R, Preece D, Campitelli G, Scott-Pillow G. The assessment of emotional reactivity across negative and positive emotions: Development and validation of the Perth Emotional Reactivity Scale (PERS). Assessment. 2017 2 1:868–879. 10.1177/1073191117694455 29214846

[21] Ripper CA, Boyes ME, Clarke PJ, Hasking PA. Emotional reactivity, intensity, and perseveration: Independent dimensions of trait affect and associations with depression, anxiety, and stress symptoms. Pers Indiv Differ. 2018 1 15;121:93–9.

[22] Quoidbach J, Berry EV, Hansenne M, Mikolajczak M. Positive emotion regulation and well-being: Comparing the impact of eight savoring and dampening strategies. Pers Indiv Differ. 2010 10 1;49(5):368–73.

[23] American Psychiatric Association. Diagnostic and statistical manual of mental disorders. 5th ed Washington: Author; 2013.

[24] Rimes KA, Chalder T. The Beliefs about Emotions Scale: validity, reliability and sensitivity to change. J Psychosom Res. 2010 3 1;68(3):285–92. 10.1016/j.jpsychores.2009.09.014 20159215

[25] Harmon-Jones E, Harmon-Jones C, Amodio DM, Gable PA. Attitudes toward emotions. J Pers Soc Psychol. 2011 12;101(6):1332 10.1037/a0024951 21843012

[26] Halberstadt AG, Dunsmore JC, Bryant A Jr, Parker AE, Beale KS, Thompson JA. Development and validation of the Parents’ Beliefs About Children’s Emotions Questionnaire. Psychol Assessment. 2013 12;25(4):1195. 10.1037/a0033695 PMC4018216 23914957

[27] Veilleux JC, Salomaa AC, Shaver JA, Zielinski MJ, Pollert GA. Multidimensional assessment of beliefs about emotion: Development and validation of the emotion and regulation beliefs scale. Assessment. 2015 2;22(1):86–100. 10.1177/1073191114534883 24835246

[28] Gratz KL, Roemer L. Multidimensional assessment of emotion regulation and dysregulation: Development, factor structure, and initial validation of the difficulties in emotion regulation scale. J Psychopathol Behav. 2004 3 1;26(1):41–54.

[29] Little TD, Lindenberger U, Nesselroade JR. On selecting indicators for multivariate measurement and modeling with latent variables: When" good" indicators are bad and" bad" indicators are good. Psychol Methods. 1999 6;4(2):192.

[30] Stevens J. P. Applied multivariate statistics for the social sciences. NJ: Erlbaum; 2002.

[31] Lovibond PF, Lovibond SH. The structure of negative emotional states: Comparison of the Depression Anxiety Stress Scales (DASS) with the Beck Depression and Anxiety Inventories. Behav Res Ther. 1995 3 1;33(3):335–43. 10.1016/0005-7967(94)00075-u 7726811

[32] Wang CL, Ahmed PK. The development and validation of the organisational innovativeness construct using confirmatory factor analysis. European Journal of Innovation Management. 2004 12 1:7(4):303–313.

[33] Kline RB. Principles and practice of structural equation modeling. NY: Guilford; 2016.

[34] Brown TA. Confirmatory factor analysis for applied research. NY: Guilford; 2015.

[35] Kline P. Psychometrics and psychology. London: Academic Press; 1979.

[36] Gorsuch RL. Factor analysis. NJ: Erlbaum; 1983.

[37] Marsh HW, Hau KT, Wen Z. In search of golden rules: Comment on hypothesis-testing approaches to setting cutoff values for fit indexes and dangers in overgeneralizing Hu and Bentler’s (1999) findings. Struct Equ Modeling. 2004 7 1;11(3):320–41.

[38] Byrne BM. Structural equation modeling with AMOS: Basic concepts, applications, and programming. NY: Routledge; 2016.

