# Supplementary material for: Assessing beliefs about emotions: Development and validation of the Emotion Beliefs Questionnaire
Source: PLoS One. 2020 Apr 14;15(4):e0231395. doi: 10.1371/journal.pone.0231395 (PMC7156043; doi:10.1371/journal.pone.0231395)
Supplement: S1 Table — *Item retained in the 16-item EBQ. (DOCX) [file pone.0231395.s001.docx]

Table S1.
*An Ordered List of the 30 Administered EBQ Items in the Development Pool*

| Item content |
| --- |
| People cannot control their positive emotions.* |
| Negative emotions are overwhelmingly detrimental to people. |
| Negative emotions shouldn’t play a crucial part in people’s lives. |
| People don’t need their positive emotions.* |
| Positive emotions are harmful.* |
| The intensity of positive emotions is not something people can control. |
| People cannot control their negative emotions.* |
| Positive emotions are overwhelmingly detrimental to people. |
| Positive emotions shouldn’t play a crucial part in people’s lives. |
| People don’t need their negative emotions.* |
| Negative emotions are harmful.* |
| The intensity of negative emotions is not something people can control. |
| People cannot learn techniques to effectively control their positive emotions.* |
| Negative emotions are very unhelpful to people. |
| It’s not necessary to consider negative emotions in order to live a balanced life. |
| Positive emotions aren’t an important part of life. |
| There is very little use for positive emotions.* |
| It doesn’t matter how hard people try, they cannot change their positive emotions.* |
| People cannot learn techniques to effectively control their negative emotions.* |
| Positive emotions are very unhelpful to people.* |
| It’s not necessary to consider positive emotions in order to live a balanced life. |
| Once people are experiencing negative emotions, there is nothing they can do about modifying them.* |
| The presence of negative emotions is a bad thing for people.* |
| In successful relationships (with family or friends), negative emotions shouldn’t play a central role. |
| Negative emotions aren’t an important part of life. |
| There is very little use for negative emotions.* |
| It doesn’t matter how hard people try, they cannot change their negative emotions.* |
| In successful relationships (with family or friends), positive emotions shouldn’t play a central role. |
| The presence of positive emotions is a bad thing for people. |
| Once people are experiencing positive emotions, there is nothing they can do about modifying them.* |

*Note*. *Item retained in the 16-item EBQ.
