# Supplementary material for: Assessing beliefs about emotions: Development and validation of the Emotion Beliefs Questionnaire
Source: PLoS One. 2020 Apr 14;15(4):e0231395. doi: 10.1371/journal.pone.0231395 (PMC7156043; doi:10.1371/journal.pone.0231395)
Supplement: S2 Table — Factor loadings < .20 are not displayed. Principal axis factoring with direct oblimin rotation was used. Three factors were extracted (eigenvalues > 1) accounting for 62.38% of the variance in item scores. Correlations between the three factors were as follows: F1-F2 = .28, F1-F3 = .49, F2-F3 = .18. (DOCX) [file pone.0231395.s002.docx]

Table S2.
*Factor Loadings from an Exploratory Factor Analysis of the 16 Retained EBQ Items.*

| Item content | Factor 1 | Factor 2 | Factor 3 |
| --- | --- | --- | --- |
| People cannot control their negative emotions. | .72 |  |  |
| Once people are experiencing negative emotions, there is nothing they can do about modifying them. | .75 |  |  |
| It doesn’t matter how hard people try, they cannot change their negative emotions. | .70 |  |  |
| People cannot learn techniques to effectively control their negative emotions. | .81 |  |  |
| People cannot learn techniques to effectively control their positive emotions. | .52 |  | .24 |
| Once people are experiencing positive emotions, there is nothing they can do about modifying them. | .69 |  |  |
| It doesn’t matter how hard people try, they cannot change their positive emotions. | .64 |  |  |
| People cannot control their positive emotions. | .35 |  |  |
| People don’t need their negative emotions. |  | .80 |  |
| The presence of negative emotions is a bad thing for people. |  | .70 |  |
| There is very little use for negative emotions |  | .65 |  |
| Negative emotions are harmful. |  | .76 |  |
| People don’t need their positive emotions. |  |  | .70 |
| Positive emotions are harmful. |  |  | .69 |
| There is very little use for positive emotions. | .23 |  | .71 |
| Positive emotions are very unhelpful to people. |  |  | .72 |

*Note.* Factor loadings < .20 are not displayed. Principal axis factoring with direct oblimin rotation was used. Three factors were extracted (eigenvalues > 1) accounting for 62.38% of the variance in item scores. Correlations between the three factors were as follows: F1-F2= .28, F1-F3= .49, F2-F3=.18.
