# Supplementary material for: Assessing beliefs about emotions: Development and validation of the Emotion Beliefs Questionnaire
Source: PLoS One. 2020 Apr 14;15(4):e0231395. doi: 10.1371/journal.pone.0231395 (PMC7156043; doi:10.1371/journal.pone.0231395)
Supplement: S2 File — (PDF) [file pone.0231395.s004.pdf]

EBQ

Name: **Date:**

This questionnaire asks about your beliefs about emotions **in general**. Some questions ask about negative emotions (e.g., sadness, fear, and anger). Other questions ask about positive emotions (e.g., happiness, joy, and amusement). For each statement, please rate **how much you agree or disagree that the statement is true *in general***. Circle one answer for each statement.

		Strongly disagree	----	----	Neither agree nor disagree	----	----	Strongly agree
1	Once people are experiencing negative emotions, there is nothing they can do about modifying them.	1	2	3	4	5	6	7
2	People cannot control their positive emotions.	1	2	3	4	5	6	7
3	There is very little use for negative emotions.	1	2	3	4	5	6	7
4	Positive emotions are very unhelpful to people.	1	2	3	4	5	6	7
5	It doesn’t matter how hard people try, they cannot change their negative emotions.	1	2	3	4	5	6	7
6	People cannot learn techniques to effectively control their positive emotions.	1	2	3	4	5	6	7
7	People don’t need their negative emotions.	1	2	3	4	5	6	7
8	There is very little use for positive emotions.	1	2	3	4	5	6	7
9	People cannot control their negative emotions.	1	2	3	4	5	6	7
10	It doesn’t matter how hard people try, they cannot change their positive emotions.	1	2	3	4	5	6	7
11	Negative emotions are harmful.	1	2	3	4	5	6	7
12	People don’t need their positive emotions.	1	2	3	4	5	6	7
13	People cannot learn techniques to effectively control their negative emotions.	1	2	3	4	5	6	7
14	Once people are experiencing positive emotions, there is nothing they can do about modifying them.	1	2	3	4	5	6	7
15	The presence of negative emotions is a bad thing for people.	1	2	3	4	5	6	7
16	Positive emotions are harmful.	1	2	3	4	5	6	7

Emotion Beliefs Questionnaire (EBQ) Scoring Instructions

The EBQ is a 16-item self-report measure of beliefs about emotions. Based on Ford and Gross's (2019) theoretical framework, the EBQ assesses two main categories of beliefs about emotions: beliefs about the *controllability* of emotions and beliefs about the *usefulness* of emotions. These beliefs are assessed for negative emotions (e.g., sadness) and positive emotions (e.g., happiness).

Four subscale scores and three composite scores are designed to be derived from the measure, with higher scores indicating more maladaptive beliefs about emotions (i.e., stronger beliefs that emotions are uncontrollable and useless). The table below describes each of these scores and how to calculate them.

Subscale/composite	How to calculate	Content measured
**Subscale scores**		
Negative-Controllability	Sum items 1, 5, 9, 13.	Beliefs about how uncontrollable negative emotions are.
Positive-Controllability	Sum items 2, 6, 10, 14.	Beliefs about how uncontrollable positive emotions are.
Negative-Usefulness	Sum items 3, 7, 11, 15.	Beliefs about how useless (e.g., undesirable, unimportant, or harmful) negative emotions are.
Positive-Usefulness	Sum items 4, 8, 12, 16.	Beliefs about how useless (e.g., undesirable, unimportant, or harmful) positive emotions are.
**Composite scores**		
General-Controllability	Sum Negative-Controllability and Positive-Controllability subscales	Beliefs about how uncontrollable negative and positive emotions are.
General-Usefulness	Sum Negative-Usefulness and Positive-Usefulness subscales	Beliefs about how useless (e.g., undesirable, unimportant, or harmful) negative and positive emotions are.
Total scale	Sum all items	Overall marker of maladaptive beliefs about emotions; composite of beliefs about how uncontrollable and useless negative and positive emotions are.
